# Characteristics of *Klebsiella* spp. from sheep flocks in northern China

**DOI:** 10.3389/fmicb.2026.1995610

**Published:** 2026-10-30

**Authors:** Hai-Tao Wang, Hai-Long Yu, Li Guo, Xiang-Yu Wang, Lu-Yao Tang, Meng-Ting Yang, Wen-Xu Tan, Kai-Meng Shang, Hong-Bo Ni, Gui-zhe Yao, Ying Li, Rui Liu

**Affiliations:** 1 College of Life Sciences, Changchun Sci-Tech University, Changchun, Jilin, China; 2 College of Food and Pharmaceutical Engineering, Wuzhou University, Wuzhou, China; 3 College of Veterinary Medicine, Qingdao Agricultural University, Qingdao, Shandong, China; 4 Animal Science and Technology College, Jilin Agricultural Science and Technology University, Jilin, China; 5 College of Veterinary Medicine, Yangzhou University, Yangzhou, Jiangsu, China; 6 Heilongjiang Provincial Animal Disease Prevention and Control Center, Harbin, Heilongjiang, China

**Keywords:** antibiotic resistance, *Klebsiella* spp., sheep, virulence factors, whole genome sequencing

## Abstract

**Background:**

*Klebsiella* spp. are Gram-negative opportunistic pathogens that affect both humans and animals. However, data on their antimicrobial resistance and virulence characteristics in sheep, particularly in China, remain limited.

**Methods:**

In 2024, 421 sheep fecal samples were collected from six regions in northern China. *Klebsiella* spp. were isolated and identified, followed by antimicrobial susceptibility testing and PCR screening for virulence-associated genes. The pathogenic potential of selected isolates was evaluated using the *Galleria mellonella* infection model. Whole-genome sequencing was performed on 26 selected isolates to characterize their antimicrobial resistance and virulence determinants.

**Results:**

*Klebsiella* spp. were isolated from 120 samples, corresponding to an overall detection rate of 28.5%, including 85 *Klebsiella pneumoniae* isolates (70.8%, 85/120). All isolates were resistant to sulfamethoxazole, with high resistance to tetracycline (76.7%), florfenicol (42.5%), and ciprofloxacin (41.7%). Resistance to tigecycline (10.0%) and polymyxin B (5.8%) was also observed at lower levels. Overall, 64.2% of the isolates exhibited multidrug resistance. PCR screening revealed virulence-associated genes, including *iucA* (10.8%), *rmpA* (8.3%), *peg*-344 (0.8%), and *iroB* (3.3%). Based on their virulence-associated gene profiles, 14 isolates were selected for further analysis, of which 11 exhibited high pathogenic potential in the *Galleria mellonella* infection model. Whole-genome sequencing revealed diverse virulence and antimicrobial resistance determinants. No plasmid-mediated resistance determinants related to polymyxin B (e.g., mcr-like genes) or recognized tigecycline resistance determinants were identified in the genomes. In the isolates analyzed in this study, mutations in lipid A modification and regulatory genes, including *arnT* and *pmrA*/*pmrB*, may have contributed to polymyxin B resistance, while mutations in efflux pump-associated genes, including *oqxA*/*oqxB, acrA*, and *acrR*, may have contributed to tigecycline resistance.

**Conclusion:**

Multidrug resistance and pathogenic potential coexist among *Klebsiella* spp. isolated from healthy sheep in northern China. These findings suggest that sheep-associated *Klebsiella* may serve as reservoirs of clinically relevant antimicrobial resistance and virulence determinants, highlighting the need for continued surveillance and prudent antimicrobial use in livestock.

## Introduction

*Klebsiella* spp. are Gram-negative, non-motile, rod-shaped bacteria found in diverse environments and in the gastrointestinal tract of humans and animals (Denissen et al., 2022; Shehata et al., 2024; Arafa, 2024; Hameed et al., 2026). In humans, *Klebsiella pneumoniae* can cause pneumonia, septicemia, or wound infections, accounting for an estimated 800,000 deaths annually, most of which are associated with antimicrobial resistance (AMR) (Song et al., 2025). In recent years, outbreaks of antimicrobial-resistant *Klebsiella* strains have been reported worldwide (Chu et al., 2024; Li et al., 2025; Yang et al., 2026).

Sheep are important livestock animals that contribute to the production of meat, milk, wool, and other animal products (Simões et al., 2021; Bhatt and Abbassi, 2021). However, infectious diseases, including respiratory disease, mastitis, enteric disease, and footrot, which can cause substantial economic losses in sheep production (Nielsen et al., 2021; Su et al., 2026). These diseases are associated with diverse bacterial pathogens, such as *Mannheimia haemolytica, Pasteurella multocida, Staphylococcus spp*., *Escherichia coli*, and *Dichelobacter nodosus* (Gelasakis et al., 2015; Zanolari et al., 2021; Simões et al., 2021). *Klebsiella* spp. have also been associated with mastitis and pneumonia in sheep, with reported prevalence ranging from 1.6% in healthy animals to over 50% in diseased populations (Madhi et al., 2024; Liu S. et al., 2026). Given the increasing number of reports on hypervirulent *Klebsiella* strains and their zoonotic potential, it is essential to characterize the virulence profiles of *Klebsiella* isolates from sheep (García-Cobos et al., 2025).

AMR is a major global health threat, and the extensive use of antibiotics in livestock production contributes to the selection and dissemination of resistant bacteria (Denissen et al., 2022). The emergence of multidrug-resistant (MDR) and extensively drug-resistant (XDR) strains poses significant challenges to disease prevention and treatment in both veterinary and human medicine (Wang et al., 2020; Sun et al., 2026a; Ahmad et al., 2024; Mubashar et al., 2025a; Wu X. et al., 2024; Mubashar et al., 2025b). Therefore, surveillance of AMR in sheep-associated bacteria is important for assessing their potential impact on animal and public health (Herawati et al., 2023).

However, the occurrence, antimicrobial resistance, and virulence characteristics of *Klebsiella* associated with healthy sheep, particularly in China, remain insufficiently characterized. Limited information is also available on the mechanisms underlying resistance to clinically important antibiotics such as polymyxin B and tigecycline. This study therefore investigated the antimicrobial resistance and virulence profiles of sheep-associated *Klebsiella* in northern China, with particular emphasis on polymyxin B and tigecycline resistance, and further characterized their genomic determinants.

## Materials and methods

### Sample collection and bacterial isolation

Between March and September 2024, 421 fresh fecal samples were collected from sheep farms across Heilongjiang (*n* = 62), Jilin (*n* = 73), Shandong (*n* = 148), Gansu (*n* = 20), Shaanxi (*n* = 80), and Inner Mongolia Autonomous Region (*n* = 38), China. *Klebsiella* was selectively isolated from the samples using MacConkey agar supplemented with inositol, adonitol, and carbenicillin (MIAC). Suspected *Klebsiella* colonies were initially identified based on colony morphology and further confirmed by 16S rRNA gene sequencing, with sequences compared to those in the NCBI database (Supplementary Table 1). The 16S rRNA gene sequences obtained in this study have been deposited in GenBank under accession numbers PZ292769-PZ292783.

### Antibiotic resistance phenotypic characterization

Antimicrobial susceptibility testing was performed in accordance with the guidelines of the European Committee on Antimicrobial Susceptibility Testing (EUCAST; https://www.eucast.org/). The antimicrobial panel comprised 14 antibiotics, including gentamicin, amikacin, polymyxin B, cefotaxime, ceftazidime, meropenem, imipenem, tetracycline, tigecycline, enrofloxacin, ciprofloxacin, florfenicol, azithromycin, and sulfisoxazole. Minimum inhibitory concentrations (MICs) were determined using the agar dilution method following standard procedures. The MIC of polymyxin B was determined using the broth microdilution method recommended by EUCAST. The MIC results were interpreted according to the EUCAST Clinical Breakpoints Table (v14.0) (2024). *Escherichia coli* ATCC 25922 was used as quality control.

### Screening of *Klebsiella* isolates with high pathogenic potential

The hypermucoviscous phenotype was assessed using the string test, as previously described. Briefly, a single colony grown overnight on blood agar plates was stretched using an inoculation loop, and isolates producing a continuous mucoviscous string >5 mm were considered hypermucoviscous (HMV)-positive (Fang et al., 2004).

To determine capsular serotypes, the *wzi* gene was amplified by PCR using primers (Supplementary Table 1), and the resulting amplicons were sequenced. Capsular serotypes (K-types) and capsular loci (KL-types) were then determined via the BIGSdb-Pasteur database (https://bigsdb.pasteur.fr/klebsiella/) (Brisse et al., 2013).

PCR amplification of the 16 virulence genes in *Klebsiella* isolates was performed using primers previously described and are listed in Supplementary Table 1 (Compain et al., 2014; Jian-Li et al., 2017; Mai et al., 2023). The PCR products were separated by electrophoresis on 1% agarose gels in 1 × TAE buffer, stained with ethidium bromide, and visualized under UV illumination. Appropriate positive and negative controls were included for each PCR run.

Based on the results of the string test, *wzi* genotyping, and virulence-associated gene screening described above, isolates showing potentially enhanced virulence-associated characteristics were selected for further evaluation in the *Galleria mellonella* infection model (Insua et al., 2013). The selection criteria were based on previously reported virulence-associated characteristics, including K1 or K2 capsular serotypes, a positive hypermucoviscous phenotype, and the presence of at least two virulence-associated genes among *iucA, rmpA, peg-344*, and *iroB* (Khairuddin et al., 2025).

### Galleria mellonella infection assay

Each isolate was injected into 10 *G. mellonella* larvae via the last left proleg at doses of 1 × 10^4^, 1 × 10^5^, and 1 × 10^6^ CFU per larva. Each dose group consisted of 10 larvae per independent experiment, and the experiment was independently repeated three times. PBS-injected and untreated larvae were included as control groups. Larval survival was monitored for 72 h, and survival data were analyzed using the Kaplan-Meier method. Survival curves were compared among groups using the log-rank (Mantel-Cox) test. Each individual larva was considered an experimental unit. Isolates causing ≥80% mortality within 72 h at an inoculum of 1 × 10^5^ CFU per larva were defined as hypervirulent, as previously described (Mai et al., 2023).

### Whole-genome sequencing and analysis

A total of 26 selected *Klebsiella* isolates were subjected to whole-genome sequencing, including isolates showing tigecycline or polymyxin B resistance and isolates with selected virulence-associated characteristics indicative of high pathogenic potential. Genomic DNA was extracted from overnight *Klebsiella* cultures using a TIANamp Genomic DNA Kit (DP304; Tiangen, Beijing, China) according to the manufacturer's instructions. Paired-end sequencing libraries were prepared and sequenced on the Illumina platform (Illumina, San Diego, CA, USA).

Raw sequencing data were quality controlled using FASTP (v0.24.0) (Chen et al., 2018; Ni et al., 2026; Liu J. et al., 2026; Zhang Y. F. et al., 2026) to remove low-quality reads and adapter contamination, generating high-quality clean reads for subsequent genome assembly and analysis. *De novo* assembly was performed using SPAdes (v3.1.0) (Bankevich et al., 2012), and the assembly gaps were closed using GapCloser (v1.12) (Luo et al., 2012). Assembly completeness and contamination were evaluated using CheckM2 (v1.0.1) (Chklovski et al., 2023; Zhang X. X. et al., 2026; Yu et al., 2026), while QUAST (v5.2.0) (Gurevich et al., 2013; Wang et al., 2026) was used to assess genome quality metrics, including contig number, N50, N90, GC content, and genome length.

Gene annotation was performed using Prokka (v1.14.6) (Seemann, 2014). Multilocus sequence typing (MLST) was performed using Kleborate (v2.2.0) (Lam et al., 2021). Acquired ARGs and mobile genetic elements (MGEs) were identified using the Center for Genomic Epidemiology (CGE) tools ResFinder (v4.1) and MobileElementFinder (v1.0.3) (Stein et al., 2024; Bortolaia et al., 2020; Camacho et al., 2009; Shi et al., 2025) Predicted protein sequences were compared against the Virulence Factor Database (VFDB) (Chen et al., 2005; Sun et al., 2026b) using DIAMOND (v2.1.11) (Buchfink et al., 2015; Shang et al., 2025), applying thresholds of ≥80% identity, ≥80% query coverage, bit score ≥60, and E-value ≤ 1e−5.

Plasmid-mediated resistance genes, including *mcr*-like and *tmexCD-toprJ*-like clusters, were screened using the ResFinder tool via CGE. BLAST analyses (NCBI BLAST+ v2.17.0) were performed to query the protein and nucleotide sequences of known resistance-associated genes against the assembled genomes. Particular focus was placed on chromosomal loci involved in polymyxin B resistance (e.g., *arnT, PmrA, PmrB, MgrB, PhoP, PhoQ, LpxA, LpxC, LpxD*) and efflux pump-associated genes linked to tigecycline resistance (e.g., *AcrAB-TolC, OqxAB, RamR, AcrR*). Mutations in these loci have been reported to contribute to reduced susceptibility to polymyxin B or tigecycline through altered lipid A structure or increased efflux pump expression (Pitt et al., 2018; Serwy et al., 2026; Dwibedy et al., 2026; Xu et al., 2025).

### Genome sequence data availability

The whole-genome sequencing data generated in this study have been deposited in the NCBI BioSample database under accession numbers SAMN54738933-SAMN54738958 and are associated with BioProject PRJNA1405173.

### Data visualization

Phylogenetic trees were visualized and annotated using iTOL (https://itol.embl.de). Network analysis and visualization were performed using the Gephi software (v0.10.1). All remaining figures were generated using R software (v4.4.1) and GraphPad Prism (v10.1.2).

## Result

### Isolation and identification results of *Klebsiella*

A total of 421 fresh fecal samples were collected from sheep across six provinces in northern China, from which 120 *Klebsiella* isolates were recovered, corresponding to an overall isolation rate of 28.5%. The isolation rates varied among regions, with the highest rate observed in Inner Mongolia (89.5%), followed by Shandong (31.8%), Gansu (25.0%), Shaanxi (20.0%), and Jilin (16.4%), whereas the lowest rate was observed in Heilongjiang (9.7%). The isolates were identified as six *Klebsiella* species, with *K. pneumoniae* being the predominant species, accounting for 70.8% (85/120) of all isolates. Other identified species included *K. variicola* (15.0%, 18/120), *K. oxytoca* (7.5%, 9/120), *K. pasteurii* (4.2%, 5/120), *K. michiganensis* (1.7%, 2/120), and *K. grimontii* (0.8%, 1/120) (Table 1).

**Table 1:** Locations and numbers of *Klebsiella* isolates collected, and their species distribution.

Location	Sample number	*K. pneumoniae*	*K. variicola*	*K. oxytoca*	*K. pasteurii*	*K. michiganensis*	*K. grimontii*
Shandong	148	46	1	0	0	0	0
Inner Mongolia	38	12	11	7	2	1	1
Shaanxi	80	14	0	0	2	0	0
Jilin	73	4	5	2	1	0	0
Heilongjiang	62	5	1	0	0	0	0
Gansu	20	4	0	0	0	1	0
Total	421	85	18	9	5	2	1

### Phenotypic profiles of resistance

All the isolates were resistant to sulfamethoxazole (100%). High resistance to tetracycline (76.7%) was observed, whereas resistance to tigecycline was relatively low (10%). Resistance to chloramphenicol-class antibiotics was moderate, with 42.5% of the isolates resistant to florfenicol. The resistance rates to enrofloxacin and ciprofloxacin were 18.3% and 41.7%, respectively. The resistance rates were 24.2% for cefotaxime and only 2.5% for ceftazidime. Among the carbapenems, none of the isolates exhibited resistance to meropenem, while 22.5% were resistant to imipenem. For aminoglycosides, resistance to gentamicin and amikacin was 6.7% and 0%, respectively. In addition, 5.8% of the isolates were resistant to polymyxin B and 9.2% were resistant to azithromycin (Figure 1).

**Figure 1:**
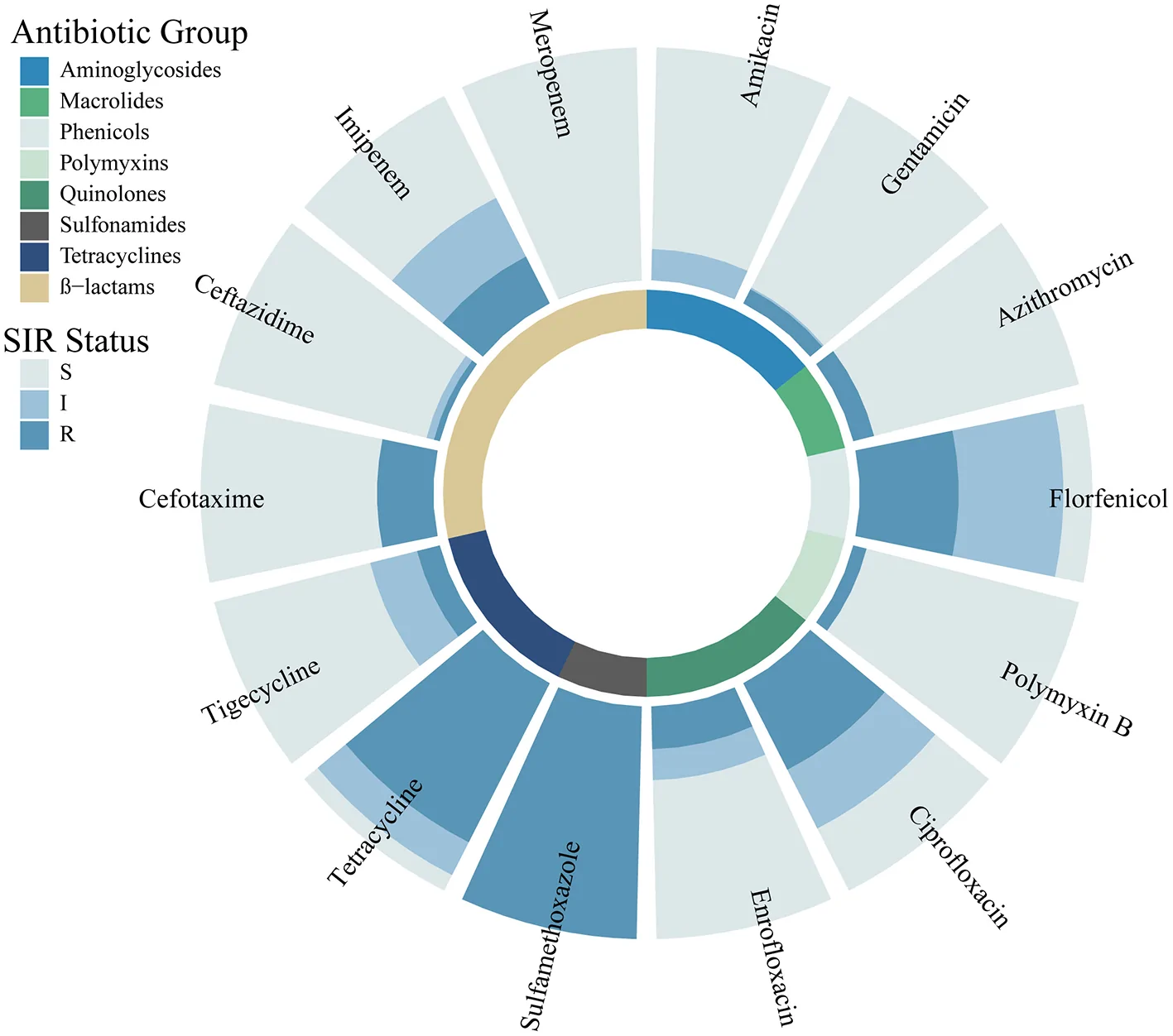
Antimicrobial Resistance of *Klebsiella* Isolates. Circular stacked characterization of antimicrobial susceptibility profiles among *Klebsiella* spp. isolates.

Statistical analysis of AMR revealed that 77 isolates (64.2%) were MDR and exhibited resistance to three or more classes of antibiotics. Among these isolates, 10 (8.3%) were further classified as XDR. These XDR isolates included nine *K. pneumoniae* and one *K. variicola* (Figure 2).

**Figure 2:**
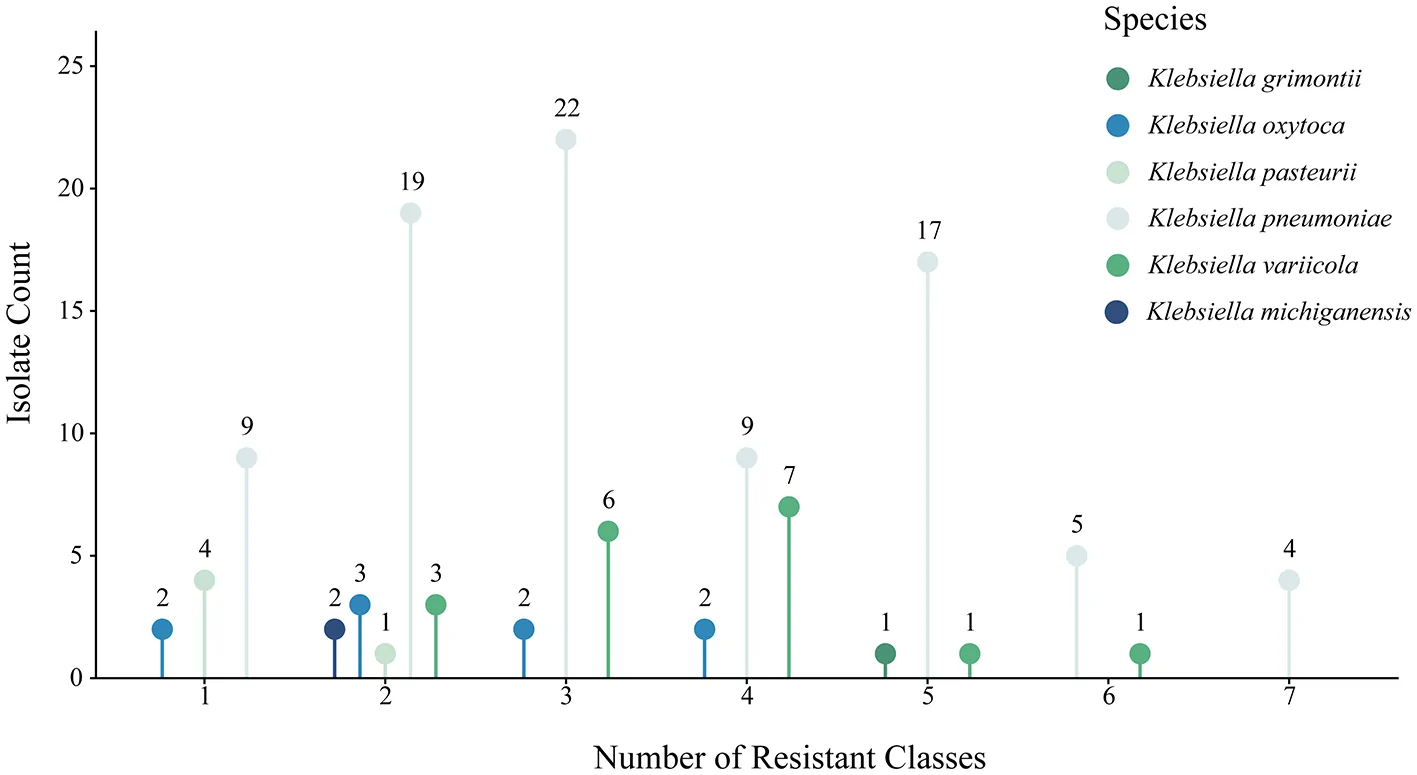
Multidrug resistance profiles of *Klebsiella* species. The lollipop chart shows the number of isolates (y-axis) categorized by the number of antibiotic resistant classes (x-axis). Colors represent different *Klebsiella* species as indicated in the legend.

### Capsular serotype distribution of *Klebsiella* isolates

Assignment to KL types was achieved for 99 isolates (Supplementary Table 2). Among them, 44 isolates matched 25 known K serotypes, most of which belonged to *K. pneumoniae*. The remaining isolates could not be assigned to any known K serotypes. The most prevalent capsular type was K22.37, followed by K12/K29 and K27 (Figure 3).

**Figure 3:**
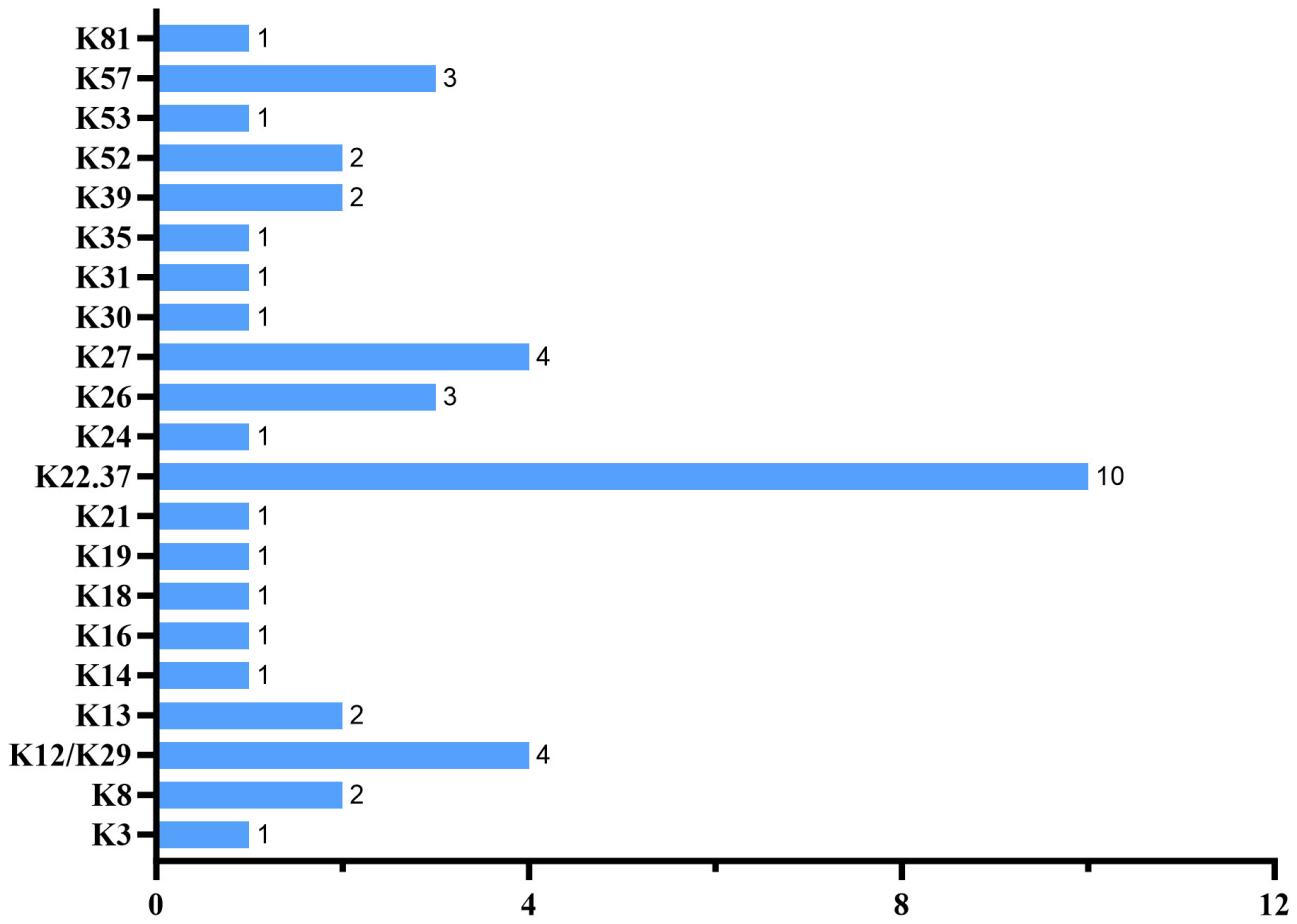
Distribution of capsular K types among *Klebsiella* isolates. The horizontal bar chart shows the number of isolates (x-axis) assigned to each capsular K type (y-axis) based on *wzi* typing.

### Virulence-associated genotypes

PCR amplification was performed to detect virulence-associated genes in the *Klebsiella* isolates. Among these isolates, genes involved in iron acquisition were detected, including *kfu* (39/120), *iutA* (27/120), *iutA2* (1/120), *iucA* (13/120), *entB* (118/120), *iroB* (4/120), and *ybtA* (13/120), with different occurrence rates among isolates. The fimbrial genes *mrkD* and *fimH* were detected in 86.7–90% of isolates, while the lipopolysaccharide biosynthesis-related genes *wabG* and *uge* were found in 95–96.7% of isolates. Additionally, 17 isolates were positive for the urease-associated gene *allS* (Supplementary Table 2).

Most *Klebsiella* isolates carried *entB, mrkD, fimH, wabG*, and *uge*, whereas *K. oxytoca* and *K. pasteurii* harbored fewer virulence genes. *rmpA* was identified in 10 isolates, including *K. pneumoniae* (n = 8), *K. variicola* (n = 1), and *K. oxytoca* (*n* = 1). The *peg-344* and *iutA2* genes were exclusively detected in *K. pneumoniae* (Figure 4).

**Figure 4:**
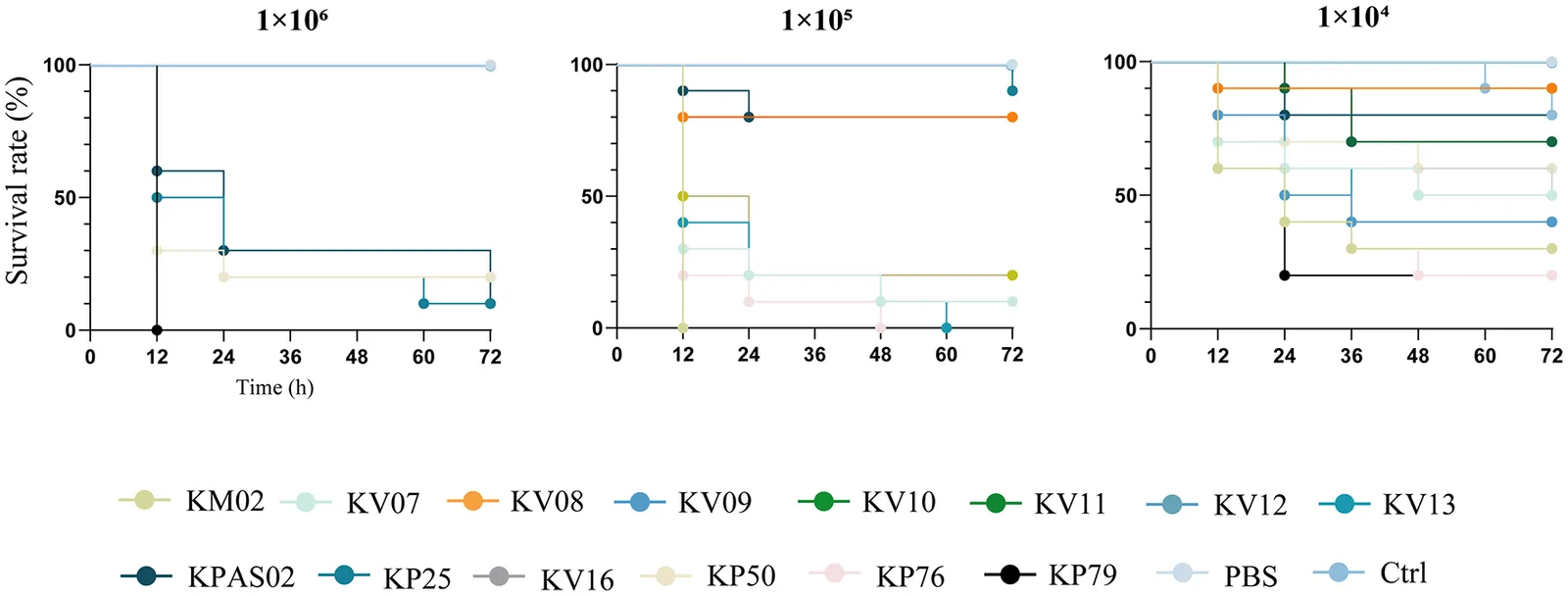
Survival of *Galleria mellonella* larvae infected with *Klebsiella* isolates. Survival curves represent the survival proportions of larvae over time following infection at three distinct inoculum levels.

### Virulence in *G. mellonella* larvae

No isolates were positive in the string test, and no isolates were identified as capsular serotypes K1 or K2. Therefore, based on the presence of at least two of the four virulence-associated genes (*iucA, rmpA, peg-344*, and *iroB*), 14 isolates were selected for further evaluation using the *Galleria mellonella* larval infection model. Of these, 11 isolates exhibited marked virulence in the larval assay. When the bacterial dose was reduced from 1 × 10^6^ CFU to 1 × 10^5^ CFU, KV08, KPAS02, and KP25 showed reduced virulence, whereas the remaining isolates still induced >70% mortality. Even at 1 × 10^4^ CFU, KM02, KV09, KV13, KP76, and KP79 remained highly lethal, causing 60–80% mortality (Figure 5).

**Figure 5:**
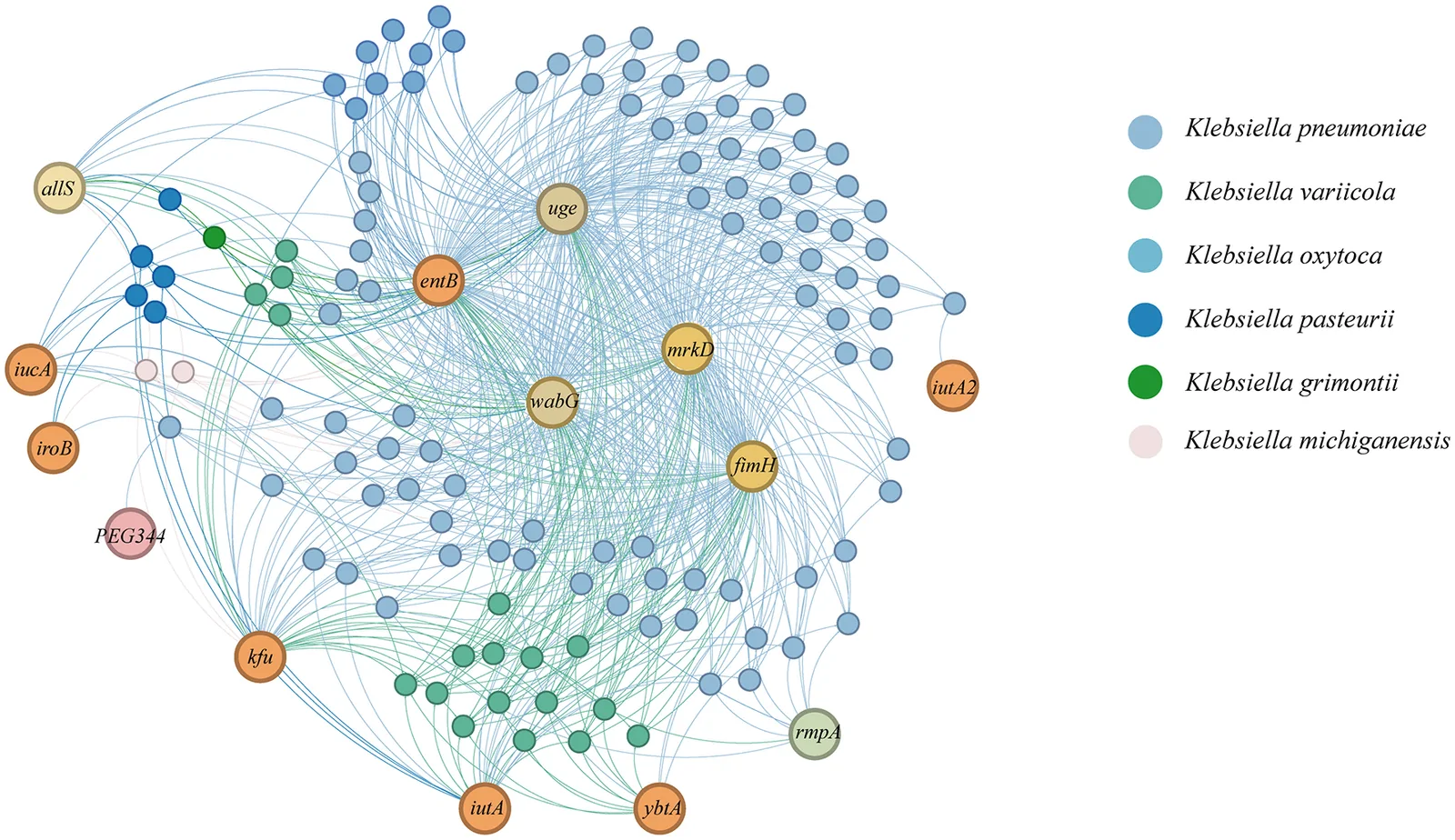
Co-occurrence network analysis of virulence factors among *Klebsiella* species. The network illustrates the correlations and co-occurrence patterns between different virulence genes.

### Genomic features and assembly quality

Genomic features of the 26 selected *Klebsiella* isolates were assessed after quality filtering of raw WGS reads using FASTP. Assemblies were of high quality, with genome sizes averaging 5.47 Mb and GC content ranging from 55.8% to 57.7% (Figure 6A). Contiguity metrics indicated robust assemblies (mean N50 = 534.4 kb; mean N90 = 155.2 kb, Figure 6B), with all genomes showing 100% completeness and minimal contamination (<1%, Supplementary Table 3).

**Figure 6:**
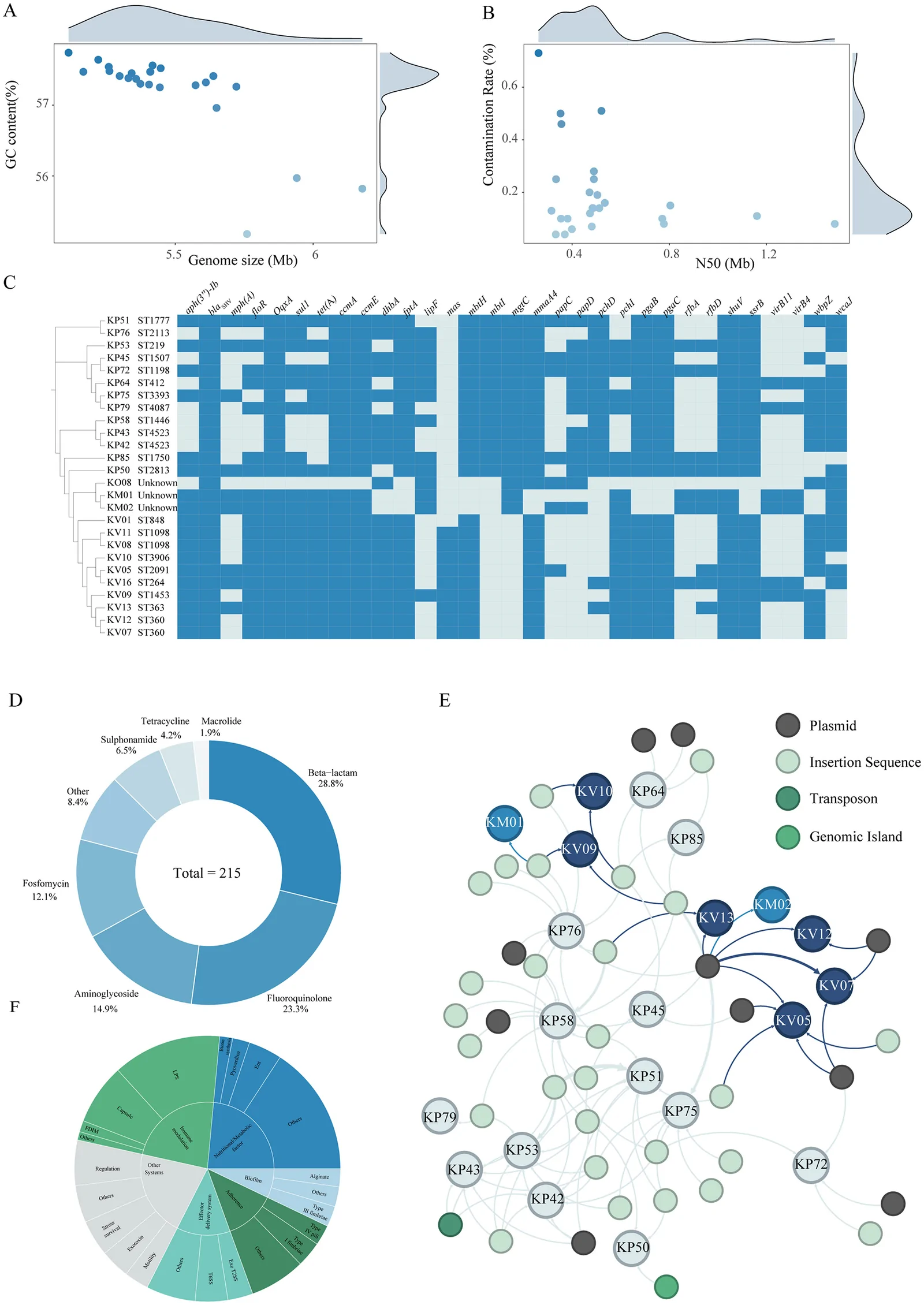
Comprehensive genomic landscape and molecular profiling of 26 Klebsiella isolates. **(A)** Joint plot of genome size vs. GC content. **(B)** Joint plot of assembly N50 vs. contamination rate. **(C)** Genomic landscape, MLST diversity, and prevalence of virulence and resistance genes among 26 *Klebsiella* spp. isolates. **(D)** Distribution of dominant ARGs identified via the CGE database. **(E)** Network analysis of genomic associations between *Klebsiella* isolates and MGEs. **(F)** Functional classification of virulence factors in *Klebsiella* isolates based on the VFDB database.

### Genetic relatedness and clustering of isolates

Analysis of genetic relatedness revealed that *K. pneumoniae* isolates clustered into three major clades. *K. oxytoca* and *K. michiganensis* were closely related and formed a distinct cluster separate from *K. variicola*. Additionally, *K. variicola* exhibited relatively high intra-species genetic diversity. Isolates belonging to the same sequence type (ST) exhibited highly similar AMR and virulence gene profiles, as exemplified by KP42 and KP43 (ST4532) (Figure 6C).

### Antimicrobial resistance gene identification

A total of 215 ARGs spanning 11 resistance classes were identified using the CGE platform (Supplementary Table 4). β-lactam resistance genes, represented by *bla*_SHV_, *bla*_TEM_, and *bla*_CTX − M_. Fluoroquinolone resistance genes included *qnrB* and *qnrS* and aminoglycoside resistance genes, including *aph(3*′′*)-Ia, aph(6*′*)-Id*, and *aadA*. Resistance genes for sulphonamides, tetracyclines, macrolides, and fosfomycin were also detected, including *sul1, sul2, tet*(A), *mphA*, and various *fosA* genes. The overall ARG profiles were largely consistent with the PCR results, with β-lactam genes being predominant (Supplementary Figure 6D).

### Mobile genetic elements analysis

In the CGE-based analysis, 106 MGEs were identified across *Klebsiella* isolates (Supplementary Table 5), including 81 non-plasmid elements and 25 plasmids. Non-plasmid MGEs were predominantly insertion sequences, with IS903, ISKpn1, and ISSty2 being the most frequent, whereas composite transposons and a single integrative mobilizable element were rare. Among the plasmids, IncFIB(K) was the most common, followed by IncR and IncFII(K), with other types occurring less frequently. Some isolates harbored multiple MGEs in combination with plasmids. For instance, KP51 carried 13 MGEs along with one plasmid (IncR), KP58 contained 12 MGEs and three plasmids (IncFIB[K], IncHI1B, and IncFIB[Mar]), and KP76 contained 13 MGEs together with two plasmids (IncHI1B and IncFIB[K]) (Figure 6E).

### Virulence factor genes analysis

Virulence gene annotation was performed for 26 *Klebsiella* isolates, resulting in the identification of 448 non-redundant virulence genes that were classified into 159 virulence factors. These factors were further grouped into 14 functional categories, including adhesion and invasion, secretion systems, and toxins (Figure 6F).

Multiple virulence-associated determinants were detected among the isolates, including siderophore-related genes (*fepABCDG* and *iroCEN*), secretion system-associated genes (*exeCDEFGHIJKL*), type I fimbriae genes (*fimABCDEFGHIK*), type III fimbriae genes (*mrkABCDEFGH*), lipopolysaccharide biosynthesis genes (*lpxABCDHK*), and O-antigen biosynthesis genes (*wcbKNRT*) (Supplementary Table 6).

### Polymyxin B resistance mechanism

Plasmid-mediated colistin resistance genes (*mcr*-like genes) were not detected in any of these isolates. Comparative analysis of polymyxin B-resistant strains, performed using BLAST against the susceptible reference strain KP79, revealed multiple mutations in key resistance-associated genes. Both resistant strains carried substitutions in *arnT*, while one strain harbored additional changes in *PmrA* and *PmrB*. Other regulatory and lipid A modification genes were conserved (Table 2) (WT, wild-type, indicates sequences identical to those of the susceptible reference strain).

**Table 2:** Mutations in polymyxin B resistance-associated genes in two polymyxin B-resistant *Klebsiella pneumoniae* strains relative to the susceptible reference strain KP79.

Sample	MIC(POLB)	*PmrA*	*PmrB*	*MgrB*	*PhoP*	*PhoQ*	*LpxA*	*LpxC*	*LpxD*	*arnB*	*arnT*
KP76	16 μg/mL	M66I	L213M	WT	WT	WT	WT	WT	WT	WT	M144L; V177I; Q156H; T335A; R372K; G403D; E441D; G503D
KP85	4 μg/mL	WT	WT	WT	WT	WT	WT	WT	WT	WT	M144L; G403D; E441D; G503D

All four polymyxin B-resistant KV strains carried the N13H substitution in *PmrB* relative to the susceptible strain KV07. Additional mutations were detected in *PmrB* (A170S, E272K), *MgrB* (R92K), *PhoQ* (N77S), *LpxC* (A135V), and *ArnT* (R5S, G405C, S446F) in a subset of the strains, whereas other resistance-associated genes remained unchanged (Table 3).

**Table 3:** Mutations in polymyxin B resistance-associated genes in four polymyxin B-resistant *Klebsiella variicola* strains relative to the susceptible reference strain KV07.

Sample	MIC(POLB)	*PmrA*	*PmrB*	*MgrB*	*PhoP*	*PhoQ*	*LpxA*	*LpxC*	*LpxD*	*arnB*	*arnT*
KV01	4 μg/mL	WT	N13H; E272K	WT	WT	WT	WT	WT	A135V	A21V	WT
KV05	4 μg/mL	WT	N13H; A170S	WT	WT	R92K	V21I	WT	A135V	WT	R5S; G405C
KV08	4 μg/mL	WT	N13H;	WT	WT	N77S; R92K	WT	WT	WT	WT	S446F
KV10	4 μg/mL	WT	N13H; A170S; E272K	WT	WT	R92K	WT	WT	A135V	WT	WT

Compared with the polymyxin B-susceptible reference strain JKo3, KO08 exhibited numerous amino acid substitutions, including multiple changes in *PmrB, PhoQ, ArnT*, and *LpxD*, along with single substitutions in *PmrA, MgrB, PhoP, LpxA, LpxC*, and *ArnB* (Table 4).

**Table 4:** Mutations in polymyxin B resistance-associated genes in the KO08 strain relative to the susceptible reference strain JKo3.

Gene	Sample KO08 POLB MIC = 4 μg/mL
*PmrA*	V48M; D86E; V129I
*PmrB*	20 amino acid substitutions
*MgrB*	T10I
*PhoP*	N145D
*PhoQ*	12 amino acid substitutions
*LpxA*	S11T; D16E; A241T
*LpxC*	E148K
*LpxD*	Q35K; D38H; V53A; R103S; N114T; A128V; N163D
*arnB*	T46A; G73S; L100V; I128V; G146A; H257A; V282A; A283V; E341D
*arnT*	18 amino acid substitutions

### Tigecycline resistance mechanism

Through CGE analysis, the KM01 strain was found to harbor a single amino acid substitution in the regulatory gene, *acrR* (F204L). No plasmid-mediated tigecycline resistance genes were detected in the isolates. Nine tigecycline-resistant *K. pneumoniae* strains exhibited mutations in several efflux pump-associated genes with recurrent substitutions in *oqxB* and variations in *oqxA*. Several strains also carried substitutions in *tolC* and *acrA*. However, no mutations were identified in *acrB, ramA*, or *marR* (Table 5).

**Table 5:** Mutations in tigecycline resistance–associated genes in nine tigecycline-resistant *Klebsiella pneumoniae* strains relative to the susceptible reference strain KP79.

Sample	MIC(TIG)	*acrA*	*acrB*	*tolC*	*oqxA*	*oqxB*	*ramA*	*marR*	*soxR*	*soxS*	*acrR*
KP42	8 μg/mL	WT	WT	WT	A121G	W511R; S540G; V661A; E749D	WT	WT	WT	WT	WT
KP43	16 μg/mL	WT	WT	WT	A121G	W511R; S540G; V661A; E749D	WT	WT	WT	WT	WT
KP45	8 μg/mL	WT	WT	Multiple missense mutations	WT	N148G; W511R; S540G; V661A; E749D	WT	WT	WT	WT	WT
KP51	8 μg/mL	WT	WT	T481P	G347R	W511R; V661A	WT	WT	WT	WT	WT
KP53	8 μg/mL	WT	WT	Multiple missense mutations	T341I	W511R; S540G; V661A; E749D	WT	WT	WT	WT	WT
KP58	8 μg/mL	WT	WT	WT	WT	W511R; S540G; V661A	WT	WT	WT	WT	WT
KP64	16 μg/mL	A188T	WT	Multiple missense mutations	WT	W511R; S540G; V661A	WT	WT	WT	WT	WT
KP72	8 μg/mL	WT	WT	Multiple missense mutations	WT	W511R; S540G; V661A	WT	WT	WT	WT	WT
KP75	16 μg/mL	WT	WT	A477V; T482N	A346T	W511R; V661A	WT	WT	WT	WT	Q8^*^

^*^Indicates a stop codon in the amino acid sequence.

Compared with the tigecycline-susceptible reference strains, KV01, KV10, and KV13 (MIC = 8 mg/L) exhibited amino acid substitutions in several efflux pump-associated genes. KV10 carried substitutions in *AcrB* (M326L, I733V) and *OqxB* (T318S, I839V) genes. KV13 harbors substitutions in *AcrA* (A387T), *OqxA* (K308Q), and *AcrR* (A150D). No mutations were detected in *tolC, ramA, marR, soxR*, or *soxS* in either of the strains (Table 6).

**Table 6:** Mutations in tigecycline resistance-associated genes in two tigecycline-resistant *Klebsiella variicola* strains relative to the susceptible reference strain KV01.

Sample	MIC(TIG)	*acrA*	*acrB*	*tolC*	*oqxA*	*oqxB*	*ramA*	*marR*	*soxR*	*soxS*	*acrR*
KV10	8 μg/mL	WT	M326L; I733V	WT	WT	T318S; I839V	WT	WT	WT	WT	WT
KV13	8 μg/mL	A387T	WT	WT	K308Q	WT	WT	WT	WT	WT	A150D

## Discussion

*Klebsiella* spp. are opportunistic pathogens of concern in both humans and animals; however, their virulence and AMR characteristics in sheep remain largely unexplored. In the present study, tetracycline resistance was particularly high (76.7%), reflecting the intensive and prolonged use of this class in livestock (Cai et al., 2025). Resistance to ciprofloxacin and florfenicol was moderate (42.5%), suggesting that these agents are being increasingly challenged but not yet widely compromised (Sani et al., 2023; Wang et al., 2025). Notably, imipenem resistance reached 22.5% despite the minimal use of carbapenems in small ruminant practices, in line with previous reports (0–23.5%) (Mahrous et al., 2023; Kadhum et al., 2025). In the present study, 64.2% of the isolates were classified as MDR. This proportion is lower than the 84.6% reported in Iran, yet higher than the 26.7–43.9% observed in cattle from China (Cai et al., 2025; Davoudabadi et al., 2023; Wu et al., 2022).

Classical hypervirulence-associated genes were detected at low frequencies: *rmpA* in approximately 8%, *ybtA* in 11%, and *allS* in 14% of the isolates, and their distribution was scattered across the collection. This limited presence suggests that high virulence determinants are uncommon in these sheep-derived isolates, consistent with previous observations that such genes are more frequent in clinical or extraintestinal strains (Jian-Li et al., 2017; Xu et al., 2022).

WGS revealed that the 26 *Klebsiella* isolates in this study belonged to 20 distinct sequence types, indicating a high genetic diversity. ST412 is predominantly found in Asia, which suggests that this region may represent an important reservoir (Wu W. et al., 2024). Additionally, ST4523, a recently described novel variant related to the high-risk ST11 lineage, has been associated with multidrug-resistant clinical isolates in China, underlining its epidemiological significance (Jia et al., 2022).

Various plasmid replicon types have been detected, including IncFIB, IncFII, IncR, IncX4, Col440I, and IncQ1. Among these, IncFIB and IncFII are well-known conjugative plasmids that play key roles in the dissemination of β-lactamase genes and other resistance determinants in *Klebsiella* spp. (Rigatou et al., 2025; Munir et al., 2025).

In our polymyxin B-resistant isolates, no plasmid-mediated *mcr* genes or *mgrB* disruption was detected. Instead, multiple amino acid substitutions were observed in *arnT, PmrA*, and *PmrB*, suggesting that these chromosomal mutations may contribute to polymyxin B resistance. Although *mgrB* disruption has been reported as the main mechanism of polymyxin B resistance in clinical *Klebsiella* strains, our findings suggest that alternative chromosomal mutations may also contribute to polymyxin B resistance in the isolates examined in this study (Pitt et al., 2018). Mutations in the efflux pump regulatory genes *ramR* and *acrR* have been reported to contribute to tigecycline resistance in *K. pneumoniae* by modulating the expression of RND efflux systems. These findings support our observations of recurrent substitutions in efflux-associated loci in the absence of plasmid-mediated determinants (Xu et al., 2025; Sheng et al., 2014).

## Conclusion

This study highlights the presence of multidrug-resistant *Klebsiella* isolates from sheep in northern China, with a subset exhibiting high pathogenic potential. Polymyxin B and tigecycline resistance was primarily associated with chromosomal mutations. These findings underscore the importance of ongoing surveillance and antimicrobial stewardship, particularly in livestock, to control the spread of resistant and virulent *Klebsiella* strains in animal populations.

## Data Availability

The datasets presented in this study can be found in online repositories. The names of the repository/repositories and accession number(s) can be found in the article/Supplementary material.
