# Supplementary material for: Characteristics of Klebsiella spp. from sheep flocks in northern China
Source: Front Microbiol. 2026 Oct 30;17:1995610. doi: 10.3389/fmicb.2026.1995610 (PMC13759811; doi:10.3389/fmicb.2026.1995610)
Supplement: Supplementary file 1 [file Table_1.docx]

**Supplement table 1. Primers Used for Gene Amplification in *Klebsiella* Isolates.**

| Gene Category | Primer | Sequence (5' to 3') | Tm (℃) | Size (bp) |
| --- | --- | --- | --- | --- |
| Identification | 7 F | AGAGTTTGATCCTGGCTCAG | 58 | 1500 |
|  | 1510 R | GGTTACCTTGTTACGACTT |  |  |
| Capsule typing | *wzi*-F | GTGCCGCGAGCGCTTTCTATCTTGGTATTCC | 55 | 580 |
|  | *wzi*-R | GAGAGCCACTGGTTCCAGAATTACCGC |  |  |
| Capsule | *magA*-F | GGTGCTCTTTACATCATTGC | 59 | 1282 |
|  | *magA*-R | GCAATGGCCATTTGCGTTAG |  |  |
|  | *rmpA*-F | ACTGGGCTACCTCTGCTTCA | 53 | 536 |
|  | *rmpA*-R | CTTGCATGAGCCATCTTTCA |  |  |
|  | *rmpA2*-F | CTTTATGTGCAATAAGGATGTT | 56 | 447 |
|  | *rmpA2*-R | CCTCCTGGAGAGTAAGCATT |  |  |
| Iron acquisition | *kfu*-F | GAAGTGACGCTGTTTCTGGC | 57 | 797 |
|  | *kfu*-R | TTTCGTGTGGCCAGTGACTC |  |  |
|  | *iutA*-F | GGCTGGACATCATGGGAACTGG | 66 | 300 |
|  | *iutA*-R | CGTCGGGAACGGGTAGAATCG |  |  |
|  | *iutA2*-F | AATCACCTGGGGGCTGGATGCT | 56 | 683 |
|  | *iutA2*-R | CCGCACCTTCCACGCCGTAAAT |  |  |
|  | *iucA*-F | ATAAGGCAGGCAATCCAG | 58 | 239 |
|  | *iucA*-R | CGCTTCACTTCTTTCACTGACAGG |  |  |
|  | *entB*-F | ATTTCCTCAACTTCTGGGGC | 56 | 508 |
|  | *entB*-R | AGCATCGGTGGCGGTGGTCA |  |  |
|  | *iroB*-F | CCGCAAAGAGACGAACCGCCTT | 56 | 235 |
|  | *iroB*-R | CGGGCAATCCCCGCTTTGACTT |  |  |
|  | *ybtA*-F | ATGACGGAGTCACCGCAAAC | 55 | 960 |
|  | *ybtA*-R | TTACATCACGCGTTTAAAGG |  |  |
| Fimbriae | *mrkD*-F | AAGCTATCGCTGTACTTCCGGCA | 60 | 340 |
|  | *mrkD*-R | GGCGTTGGCGCTCAGATAGG |  |  |
|  | *fimH*-F | TGCTGCTGGGCTGGTCGATG | 62 | 550 |
|  | *fimH*-R | GGGAGGGTGACGGTGACATC |  |  |
| Lipopolysaccharide | *wabG*-F | ACCATCGGCCATTTGATAGA | 57 | 683 |
|  | *wabG*-R | CGGACTGGCAGATCCATATC |  |  |
|  | *uge*-F | TCTTCACGCCTTCCTTCACT | 57 | 535 |
|  | *uge*-R | GATCATCCGGTCTCCCTGTA |  |  |
| Urease-associated genes | *allS*-F | CCGAAACATTACGCACCTTT | 56 | 508 |
|  | *allS*-R | ATCACGAAGAGCCAGGTCAC |  |  |
| Inner membrane transporter | *peg-344*-F | CTTGAAACTATCCCTCCAGTC | 58 | 503 |
|  | *peg-344*-R | CTTGAAACTATCCCTCCAGTC |  |  |
